# Research Progress in Special Engineering Plastic-Based Electrochromic Polymers

**DOI:** 10.3390/ma17010073

**Published:** 2023-12-22

**Authors:** Yixuan Liu, Zhen Xing, Songrui Jia, Xiangfu Shi, Zheng Chen, Zhenhua Jiang

**Affiliations:** Key Laboratory of High-Performance Plastics (Jilin University), Ministry of Education, National & Local Joint Engineering Laboratory for Synthesis Technology of High Performance Polymers, College of Chemistry, Jilin University, Xiuzheng Road 1788, Changchun 130012, China; liuyixuan22@mails.jlu.edu.cn (Y.L.); xingzhen21@mails.jlu.edu.cn (Z.X.); jiasr21@mails.jlu.edu.cn (S.J.); shixf23@mails.jlu.edu.cn (X.S.)

**Keywords:** electrochromism (EC), electrochromic polymers, high-performance polymers (HPP), special engineering plastic

## Abstract

SPECPs are electrochromic polymers that contain special engineering plastic structural characteristic groups (SPECPs). Due to their high thermal stability, mechanical properties, and weather resistance, they are also known as high-performance electrochromic polymer (HPEP or HPP). Meanwhile, due to the structural characteristics of their long polymer chains, these materials have natural advantages in the application of flexible electrochromic devices. According to the structure of special engineering plastic groups, SPECPs are divided into five categories: polyamide, polyimide, polyamide imide, polyarylsulfone, and polyarylketone. This article mainly introduces the latest research on SPECPs. The structural design, electrochromic properties, and applications of these materials are also introduced in this article, and the challenges and future development trends of SPECPs are prospected.

## 1. Introduction

Electrochromism (EC) refers to the phenomenon of stable and reversible color changes in the optical properties (reflectivity, transmittance, absorption rate, etc.) of a material under electrochemical driving [1]. Numerous intelligent application technologies, including large-scale electrochromic screens, automatic antiglare glass rearview mirrors, smart windows, electrochromic electronic skins, and so on, have been created and implemented as a result of this intriguing feature [2,3,4,5,6]. According to their types, electrochromic materials can be categorized into two categories: inorganic and organic materials. Currently, inorganic materials widely studied and applied include metal oxides such as WO_3_, NiO, TiO_2_, MoO_3_, and Prussian blue [7,8,9,10,11,12], which not only exhibit excellent electrochromic properties but have also been widely used. However, owing to the fewer color changes, complex device processing methods, high production costs, and challenges related to preparing large-area devices, there are still multiple drawbacks that need to be addressed despite the fact that inorganic materials are widely used. In contrast, organic materials have the potential to achieve large-scale production and processing of EC devices due to their ability to be processed in solution. In addition, organic materials can achieve more color changes through simple structural regulation, demonstrating enormous development potential and application prospects in the field of electrochromism. Compared to inorganic materials, the electrochromic cycling stability of organic materials is relatively poor, which is also an important research hurdle that researchers need to break through at present. Organic materials can be further grouped into organic small molecules and polymer materials. The typical representatives of organic small molecule electrochromic materials include viologen and its derivatives, pyridine and its derivatives, metal phthalocyanines, and so on [13,14,15,16]. Polymer materials can be further divided into two categories: conjugated conductive polymers and non-conjugated polymers. Conductive polymers such as polyaniline, polythiophene, polypyrrole, etc., can undergo reversible redox reactions through electrochemical doping and dedoping, and undergo reversible changes between different colors during this process [17,18,19]. Moreover, such materials can achieve rich color changes by regulating the spectral bandgap [20]. For example, the JR Reynolds group [21,22,23] has been committed to the research of conductive polymer materials, especially for polythiophene-based electrochromic materials. The group successfully achieved full coverage of conductive polymer electrochromic devices in the visible to near-infrared spectra by adjusting the push-pull electron ability of the acceptor in the main chain D-A structure and the structure (volume and length) of the polymer alkane side chains [21]. In addition, some conjugated polymer electrochromic materials have also been successfully put into the market as products. In 2008, PEDOT was successfully applied to electrochromic windows by Professor Xu Chunye [20], which was applied to the Boeing 787 aircraft, replacing the traditional mechanical side window sunshade.

Due to its exceptional performance, a specific type of electrochromic polymer with unique engineering plastic structural features has experienced rapid development in the past decade among non-conjugated electrochromic polymer materials. Based on its structural properties, this material possesses both the typical structural units of special engineering plastics and electrochromic structural units that enable it to perform electrochromic functions. Therefore, it can be uniformly referred to as “special engineering plastic based electrochromic polymers” (SPECPs), and some of the available literature also refers to it as high-performance electrochromic polymers [24]. Special engineering plastics (SPECPs) exhibit superior tensile qualities, high temperature resistance, radiation resistance, and weather resistance compared to other electrochromic polymers. These enhanced properties are attributed to the presence of distinctive structural units in SPECPs. Therefore, these materials are considered a class of electrochromic polymers that can have broad application prospects in extreme environments. In addition, SPECPs also have prominent characteristics in color variation. Due to their non-conjugated structural materials, electrochromic films prepared based on them typically exhibit a colorless or light yellow appearance (with high light transmittance) without the need for applying an external voltage (0 V). After applying voltage, they can turn into various colors, including yellow, brown, green, blue, purple, and black. This results in a significant decrease in light transmittance, making it suitable for a wide range of practical applications that require color change.

At present, the following five major categories of SPECPs are the focus of research, which names are: polyamide-imide (PAI–ECPs), polyimide (PI–ECPs), polyamide (PA–ECPs), polyarylketones (PEK–ECPs), and polyarylsulfones (PES–ECPs). Among them, PAI–ECPs, PA–ECPs, and PA–ECPs are the most popular and earliest materials studied. They now have the fastest improvement in other electrochromic qualities in addition to having the widest diversity of color change. Compared with this, although work on polyarylsulfone and polyarylketone materials started relatively late, they can not only achieve the EC color change performance aforementioned but also have better acid alkali stability and hydrolysis stability, which can meet a wider range of application needs. Therefore, they have also been deeply studied and widely discussed. However, there is still a significant gap in the electrochromic cycling stability of the aforementioned materials compared to EC-conductive polymers, which is currently one of the urgent challenges to overcome. In addition, how to construct effective EC devices with such materials is also a key issue that needs to be addressed at this stage. In this review, we will systematically introduce the development status of SPECPs materials, sort out and summarize their inherent laws and effective strategies in structural design, then analyze existing development problems and propose solutions, and forecast the future development trend of these materials.

## 2. Special Engineering Plastic-Based Electrochromic Polymers (SPECPs)

Commonly, to construct SPECPs, electrochromic groups with electrochromic activity are introduced into the main or side chains of special engineering plastics, endowing them with electrochromic properties. Triphenylamine (TPA) and its derivatives exhibit high hole mobility and excellent electrochemical performance due to their unique free radical properties [25,26]. At the same time, based on its large free volume propeller structure, EC films prepared from non-conjugated polymers of triphenylamine often have lighter colors in the neutral state without applying voltage (as their ground state absorption band is in the ultraviolet region). Upon application of a voltage, the electrochromic film is generally able to exhibit more distinct color changes. Therefore, in the past decade or so, researchers have carried out extensive and in-depth research on SPECPs based on triphenylamine with color-changing unit structures [27].

### 2.1. Polyamide-Imide Type Electrochromic Polymers (PAI–SPECPs)

**Figure 1:**
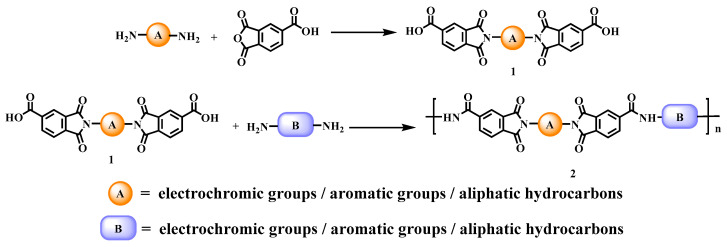
Synthetic routes to the PAIs.

Polyamide-imide type electrochromic polymers are the earliest reported special plastic-based electrochromic polymers seen in the paper [28]. PAI–ECPs are polymers that, in addition to color-changing units, also have both amide and imide structural units in the main chain of the polymer. By reacting a carboxylic acid with electrically active groups and a diamine, they first create an amide containing dicarboxylic acid. Next, they react the amide having dicarboxylic acid with another diamine that also has electrically active groups to create polyamide-imide. Figure 1 displays the structural properties and reaction formula.

**Scheme 1:**
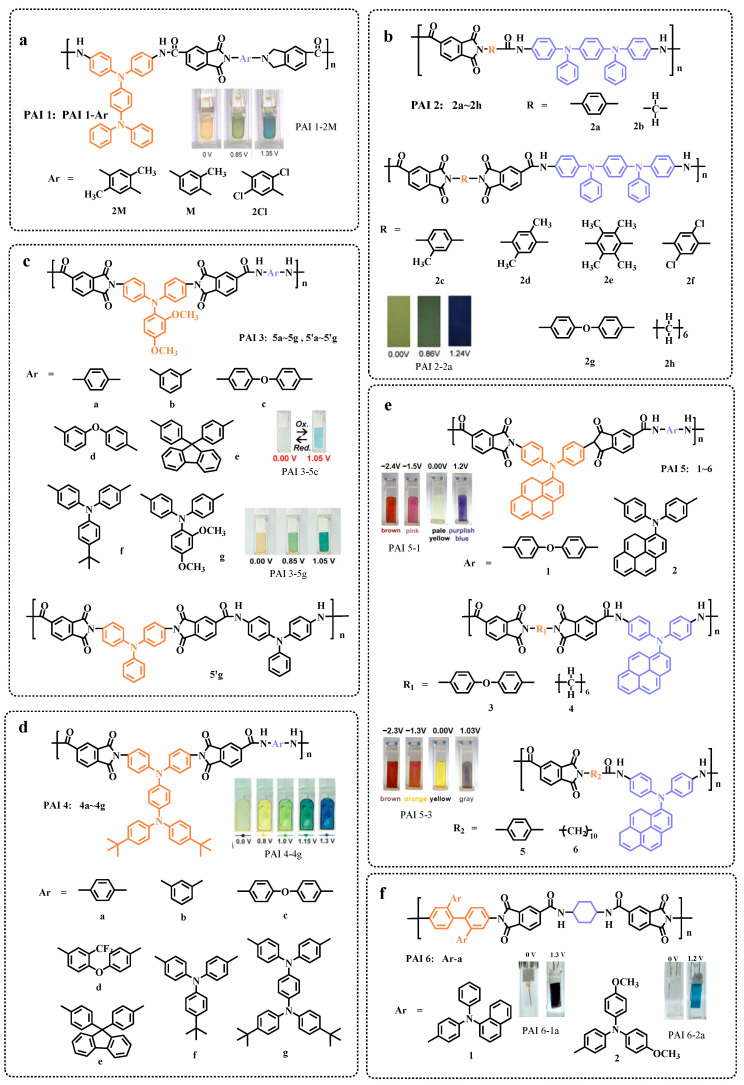
The polymers structures and color change corresponding to the PAIs. (**a**) PAI 1 series of polymers, (**b**) PAI 2 series of polymers, (**c**) PAI 3 series of polymers, (**d**) PAI 4 series of polymers, (**e**) PAI 5 series of polymers, (**f**) PAI 6 series of polymers.

In 2005, Liou’s group was the first to combine the polyamide-imide structure with the triphenylamine derivative structure (1,4-bis (diphenylamine) benzene, 1,4-bis (diphenylamine) benzene, BDPAB), and successfully prepared the PAI–1 series of polymers (PAI 1–2M, PAI 1–M, and PAI 1–2Cl, Scheme 1a) [28]. Research has shown that the introduction of the triphenylamine-based chromogenic group BDPAB not only endows polyamide-imide materials with electrochromic properties but also significantly improves the solubility of such polymers (PAI 1). These polymers not only exhibit good mechanical and electrochromic properties but also enable the electrochromic films prepared using these polymers to achieve reversible changes from a neutral state of light brown or light yellow to green and blue when subjected to voltage. Subsequently, Liou’s group developed a series of structurally similar PAI 2 polymers (PAI 2–2a~PAI 2–2h, Scheme 1b) based on this work, which also exhibited similar electrochromic properties [29]. Although the EC performance of these electrochromic polymers was not very satisfactory at that time, the photochromic properties of light to dark colors, as well as excellent mechanical properties and high temperature resistance, successfully aroused the interest of researchers in this type of material.

In 2010, Hsiao et al. reported the PAI–4 series of polymers (PAI 4–a~PAI 4–g, Scheme 1d). Since BDPAB groups have two color-changing active centers and other kinds of TPA-type color-changing groups are introduced, more color changes can be achieved in these polymers [30]. The polymer with the PAI 4–f structure exhibits reversible color changes from light yellow to yellow-green, green, and blue, while the PAI 4–g polymer exhibits stable and reversible color changes from light yellow to deep yellow, yellow-green, cyan blue, and blue.

In the same year, Hsiao et al. reported the synthesis and characterization of the PAI 3–5 series of EC polymers (PAI 3–5a~5g and PAI 3–5a′~5g′, Scheme 1c) [31]. EC films based on these polymers exhibited good electrochromic properties. Among them, because there is only one unit structure containing a single EC characteristic in the structure of PAI 3–5a~5e, their EC films can only achieve a single color reversible change from colorless (0 V) to blue (1.05 V) under voltage driving (vs. Ag/AgCl), with PAI-5c exhibiting excellent cycling stability (100 cycles). In addition, since the structure of PAI 3-5f, PAI 3–5g and PAI 3–5’g contain two different TPA color-changing units, their EC films can achieve reversible color changes between light yellow (0 V), green (0.86), and cyan (1.05 V). It is worth noting that Hsiao et al. also observed for the first time that this type of EC material can undergo reversible redox reactions under negative voltage. However, the cyclic voltammogram (CV) measurements showed that this redox behavior can only last for about five cycles, and the author did not mention any related color changes. They suggest that this negative redox characteristic is brought about by the C=O group of the imide unit in the polymer structure, so materials with this type of structure can be doped both n-type and p-type.

**Figure 2:**
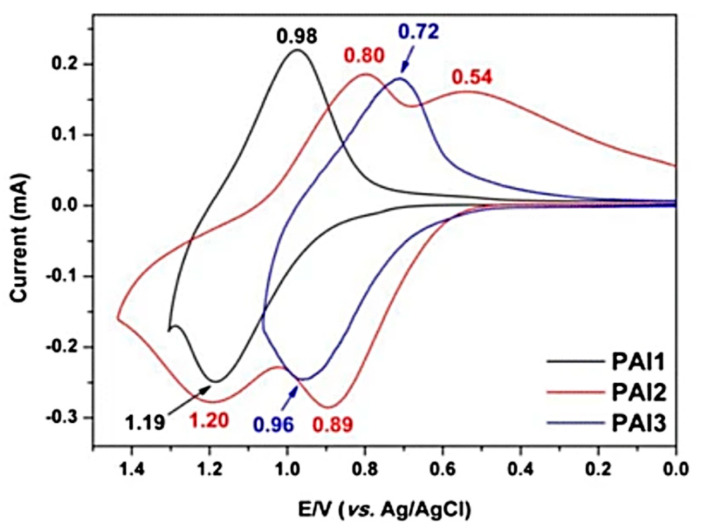
Anodic CV diagrams of the cast films of PAI1, (as well as PAI 5–1 in the main text), PAI2 (as well as PAI 5–2 in the main text) and PAI 3 (as well as PAI 5–3 in the main text) on the ITO-coated glass substrate in 0.1 M Bu_4_NClO_4_/CH_3_CN at a scan rate of 50 mV/s. Reproduced from ref. [32] with permission.

In 2015, Hsiao et al. introduced the triphenylamine-derived monomer N,N-diphenylpyren-1-amine (DPPA) into polyamide-imide polymers and successfully prepared the PAI 5 series of polymers (PAI 5–1~PAI 5–6, Scheme 1e) [32]. The effect of DPPA on electrochromic properties at different positions in the main chain was studied. The author discovered that as DPPA was introduced at different points in the main chain, the EC performance of the corresponding polymers displayed consistent variations. As shown in Figure 2, the initial potential (E_onset_) of PAI 5–3 is 0.66 V, and the peak potential of oxidation (E_pa_) is 0.96 V, while the PAI 5–1 has an initial potential of 0.90 V and a peak potential of oxidation of 1.19 V. This is due to the relatively weaker electron absorption ability of the amide unit attached to DPPA compared to the imine unit, resulting in a lower oxidation potential for PAI 5–3. Therefore, two pairs of reversible redox peaks were observed on the cyclic voltammetry curve of PAI 5–2. According to the level of potential, they correspond to the redox pairs on the amide side and the imide side, respectively. More notably, the author found that these materials exhibit bidirectional color change properties. For example, thin films based on PAI-DPPA–1 can achieve reversible color changes from white yellow (0.0 V) to purple blue (1.2 V) under forward voltage driving (vs. Ag/AgCl) and exhibit reversible color changes from white yellow (0.0 V) to pink (−1.5 V) to brown (−2.4 V) under negative voltage driving. However, the cycling stability of all materials is not ideal, and only PAI-DPPA–2 film has achieved stable reversible cycle of over 30 cycles. 

In 2019, there was a breakthrough in polyamide-imide materials. Zhang et al. designed and developed two polymers, PAI 6–1a and PAI 6–2a (Scheme 1f), that can achieve reversible changes from colorless to colored [33]. In traditional PI chain structures, the proportion of aromatic structures is high and the intermolecular hydrogen bonding force is strong, resulting in the charge transfer complex (CTC) and the PI film generally showing a yellow color. Zhang et al. effectively suppressed the formation of CTC by adding non-aromatic cyclohexane structures to the main chains of PAI 6–1a and PAI 6–2a, thereby significantly improving the transmittance of PAI 6–1a and PAI 6–2a films (both greater than 97%), making them closer to colorless under normal conditions. Among them, the EC thin film based on PAI 6–1a is colorless and transparent in a neutral state, and rapidly transforms into black after applying a voltage of 1.3 V in a three-electrode system. The EC film of PI–2a also exhibits a colorless and transparent state in a neutral state, which quickly turns blue and exhibits high EC cycle stability of 5000 cycles after applying a voltage of 1.2 V in a three-electrode system. EC materials with colorless to black transition characteristics have always been considered as the most practical and important type of material. The emergence of PAI 6–1a has shown people the enormous development potential and broad application prospects of SPECPs.

As one of the earliest developed special engineering plastic-based electrochromic polymers, the research work concerning PAI has made significant contributions to the development of this field. We summarized the performance of PAI–ECPs in Table 1, hoping to provide some inspiration for researchers by comparing these data.

### 2.2. Polyimide Type Electrochromic Polymer (PI–ECPs)

**Figure 3:**
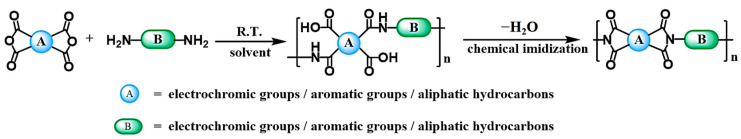
Synthetic routes to the PIs.

Polyimide-type electrochromic polymers have electrochromic groups in addition to amide units. The synthesis method used obtains the corresponding polyamic acid polymer via the ring-opening addition polymerization of the tetracarboxylic acid dianhydride type monomer and the aromatic diamine type monomer and then obtains the final target polymer via a dehydration ring-linking reaction. The preparation route is shown in Figure 3.

**Scheme 2:**
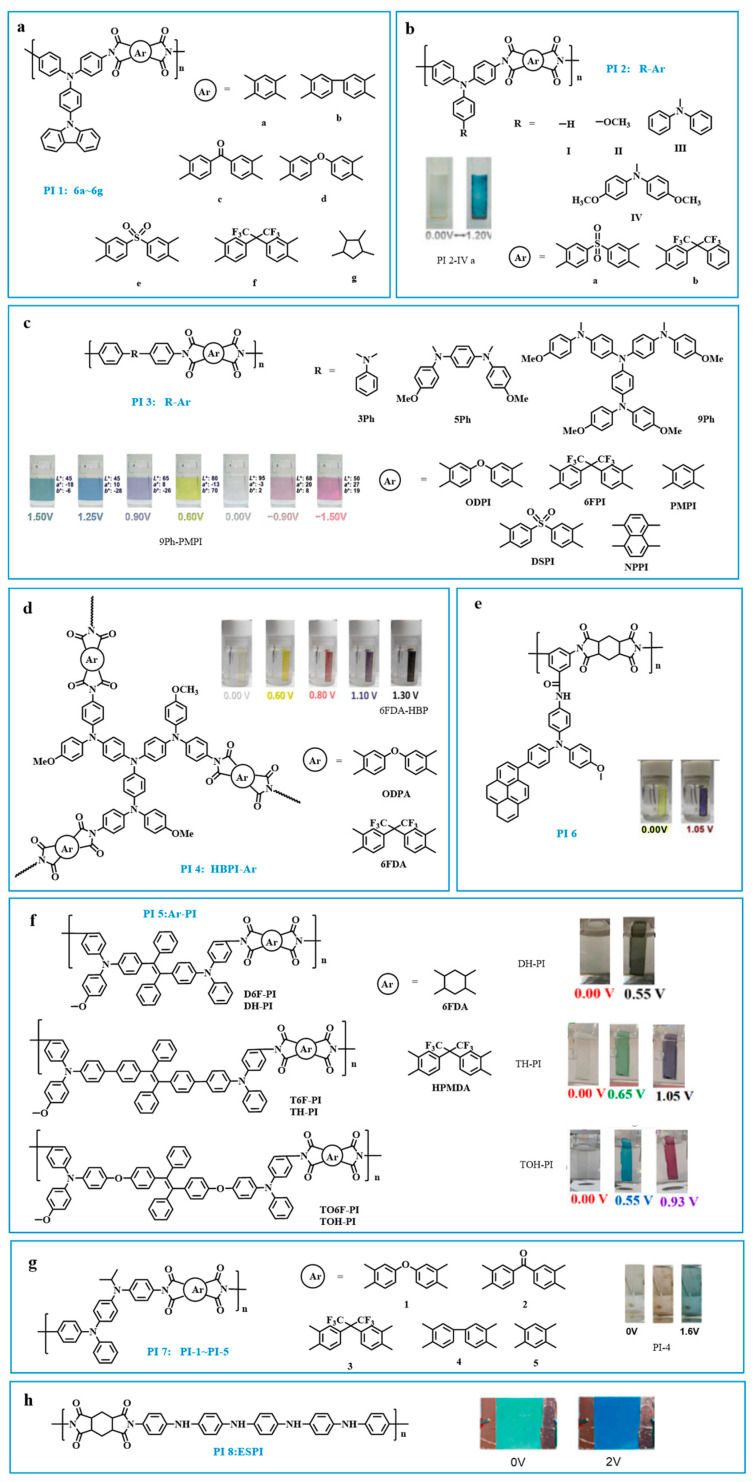
The polymer structure of (**a**) PI 1: 6a~6g (**b**) PI 2: Ia~IVa and Ib~IVb (**c**) PI 3: 3Ph~9Ph series (**d**) PI 4: HBPI (**e**) PI 5 (**f**) PI 6: PIDP (**g**) PI 7: PI-1~PI-5 and (**h**) PI 8: ESPI.

In 2006, Liou’s group introduced triphenylamine units with carbazole groups into polyimide to synthesize PI 1 series polymers 6a~6g, the structures of which are shown in Scheme 2a [34]. These polymers not only retain the high thermal stability of PI polymers (T_g_ up to 371 °C, T_d5%_ up to 607 °C) but also exhibit electrochromic properties. The EC thin films based on PI 1–6f can be changed from light yellow to green at the applied voltage of 1.15 V (vs. Ag/AgCI). Additionally, when the voltage was increased to 1.5 V, the film color turned blue. In addition, these polymers are also endowed with fluorescence characteristics due to the introduction of carbazole structural units. For example, the NMP solutions of 6f and 6g emit blue-violet fluorescence (λ_PL max_ = 431 nm) and violet fluorescence (λ_PL max_ = 402 nm) at excitation wavelengths of 308 nm and 314 nm, respectively.

In 2008, Liou’s group synthesized PI 2 series polymers (Scheme 2b) with triphenylamine derivatives [35]. It can be seen that replacing the H atom (PI 2–I) at the R1 position in the PI 2 series polymer with an electron-donating group (II, III, IV) can stabilize cationic free radicals on the TPA structure during the electrochemical oxidation doping process. This effectively prevents the tail-to-tail (para) coupling behavior of TPA between polymer chains, thereby significantly improving the EC stability of such polymers. Among them, the thin film of PI 2–IVa EC polymer can achieve reversible color change from neutral colorless to green to blue under voltage driving (vs. Ag/AgCl). In 2009, Hsiao’s group used tert-butyl to replace the H atom at the R position in PI 2–I polymer [36]. In the following years, more structural optimization and regulation research on P1 2–I was carried out by the researchers. By introducing a plurality of different substituents such as adamantane, perylene, carbazole, morpholine and viologen at the position of R, optimization and regulation of the electrochromic performance of polymers have been achieved, accumulating a lot of research experience for achieving breakthroughs in the EC performance of PI polymers [37,38,39,40,41].

In 2013, Liou’s group introduced the starburst triarylamine derived-structural unit (9Ph) with multiple N atom centers into the structure of polyimide as well as the corresponding 3Ph-series polyimides (3Ph-PIs) and 5Ph-PIs, and prepared a series of polyimide-based electrochromic polymer PI 3. The synthesized structure is shown in Scheme 2c [42]. Among them, 9Ph-6FPI is the first PI-ECP material to achieve a breakthrough in multi-color variation and high cycling stability. The color of EC thin film prepared by 9Ph–6FPI changed from colorless (L*: 96; a*: −2; b*: 3) to yellow (L*: 85; a*: −11; b*: 53), purple (L*: 69; a*: 2; b*: −8), blue (L*: 50; a*: 5; b*: −26), and then black (L*: 50; a*: −5; b*: 2). Moreover, the 9Ph–6FPI film achieved a cycling capacity of 10,000 cycles during the second oxidation stage and exhibited highly reversible CV behavior. Additionally, in the long-term stability tests of 0.60 V and 0.90 V, the yellow and purple coloring stages were maintained for 10 h, respectively, demonstrating excellent color memory effects. It is worth noting that this is the first SPECPs reported in the literature that can achieve a change from colorless to black. In the same year, the research group reported that polymer 9Ph–PMPI can achieve six color changes, breaking the record of achieving multiple color variety changes in one material once again [43]. The number of redox pairs on the cyclic voltammetry curve of electrochromic materials shows the redox reactions that the material participates in during the electrochemical process. The distribution of electrons in the polymer is modified by these redox reactions, which subsequently alters its spectral absorption. Usually, an oxidation–reduction pair represents a color change. From the point of view of molecular design, multi-color changes of polymers can be realized by increasing the redox logarithm of polymers under the condition of applying a certain voltage. The CV curve of 9Ph–PMPI has six redox pairs, with color changes in two reduction states and four oxidation states, respectively. It changes to reddish-purple (L*: 50; a*: 27; b*: 19), light pink (L*: 68; a*: 20; b*: 8), colorless (L*: 95; a*: 3; b*: 2), yellow (L*: 80; a*: 13; b*: 70), purple (L*: 65; a*: 8; b*: 26), dark blue (L*: 45; a*: 10; b*: 28) and blue-green (L*: 45; a*: 18; b*: 6) at voltages of −1.5 V, −0.90 V, 0 V, 0.60 V, 0.90 V, 1.20 V, and 1.50 V, respectively, and exhibits a relatively high optical contrast (∆T).

In 2017, Chen C.’s group successfully prepared hyperbranched PI 4–HBPI series polymers, HBPI–ODPA, and HBPI–6FPA (Scheme 2d) [44]. Thanks to the structural characteristics of hyperbranching, these two materials not only maintain the high thermal stability of PI but also achieve satisfactory solubility. In addition, due to the loose structural characteristics of hyperbranched materials, ions are easily doped and de-doped on the surface and inside of their EC thin films during EC cycling, thereby improving color switching speed and EC cycling stability. These two materials can achieve electrochromism in the visible/near-infrared region (NIR) and continuous modulation of the entire visible light region. The electrochromic performance of HBPI–6FPA is excellent, as demonstrated by its short response time (2.6 s/1.9 s at 443 nm), high optical contrast (63% at 443 nm), high coloring efficiency (196 cm^2^/C at 443 nm), excellent switching stability (5000 cycles), and long-term optical storage capacity (after the voltage is cut off for 10,000 s, the ∆T% at the 1250 nm decreases by 3%). In addition, the CV curves of HBPI thin films show four reversible redox pairs and five color transitions (from colorless to yellow, red, indigo and black). By calculating the HOMO and LUMO orbitals of triphenylamine monomers, it was found that the redox reaction occurred first in the central triphenylamine and then in the lateral triphenylamine during the reaction process. Subsequently, in 2019, Chen C.’s group constructed a novel structural unit diphenylamine pyrene (DPAP) by combining triphenylamine with fluorescent pyrene, which had EC characteristics and was introduced into PI polymers to prepare the novel EC polymer PI 6–DPAP (Scheme 2e) [45]. As a bulky drop unit along the polyimide skeleton, it improves the solubility and electrochromic/electrochromic fluorescence (EC/EFC) performance of polyimide. Although this polymer can only achieve reversible color changes from yellow to blue-purple under a driving voltage of 0~1.05 V (vs. Ag/AgCl), it exhibits a surprising EC color switching speed (tc/tb = 1.7/0.9 s), which was the fastest speed among SPECP materials at that time.

In 2022, Guan’s group developed a class of EC and EFC bifunctional polymers PI 5 containing tetraphenylethylene (TPE) units (Scheme 2f) [27]. The introduction of TPE brings fluorescence characteristics to this material. Among them, DH-PI can change color from light yellow to black at a low voltage of 0.55 V (vs. Ag/AgNO_3_). In contrast, although TH–PI can also turn black, its voltage needs to reach 1.05 V (vs. Ag/AgNO_3_).

**Figure 4:**
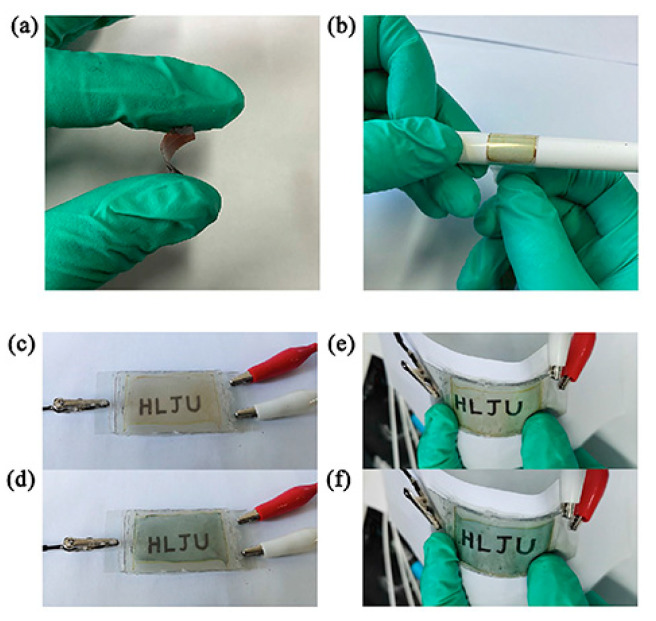
The free-standing flexible film (10 mm × 20 mm) (**a**), and the flexible PI-3/ITO/PET film (30 mm × 50 mm) (**b**). Color change of the flexible EC device (PI-3) before (**c**) and after (**d**) coloring; color change of the flexible EC device in bending state before (**e**) and after (**f**) coloring. Reproduced from ref. [46] with permission.

In 2021, Niu’s group synthesized a series of EC polymer materials PI 7 containing isopropyl side chains, the structural features of which are shown in Scheme 2g [46]. Among them, PI 7–3 can achieve reversible color changes from colorless (0 V) to orange (1.2 V) and then to blue (1.6 V) driven by voltage. After being made into a flexible device, the film cannot be brittle fractured under a certain degree of bending, and still maintains its color state after bending for 20°. The flexible electrochromic device prepared using PI 7–3 can maintain a high optical contrast stably after the cycle stability test of 1000 s (10 s once cycle), and even after undergoing a 20,000 s cycle stability test, there is no significant decrease in optical contrast (Figure 4). This work has shown people the potential application of PI–ECPs in the field of flexible devices.

In 2023, Chao’s Group introduced aniline into the polyimide backbone and prepared an electrochromic polymer ESPI, as shown in Scheme 2h. Due to aniline, ESPI exhibits excellent electrochromic performance [47]. When a voltage ranging from −0.5 V to 1.0 V is applied, the ESPI film exhibits three color variations: light yellow-green, green, and blue, with an optical contrast of 69.11% and a coloring/fading time of 0.65/1.03 s at 610 nm. Then, by employing ESPI as the working electrode and V2O5 as the counter electrode, an electrochromic device with exceptional cycling stability and memory effect can be achieved. After 10,000 cycles, the optical contrast of the device decreased by only 4%. After applying a voltage of 2 V for 20 s, the power was cut off, and after 360 s, only 1.28% attenuation was observed. Subsequently, a 0 V voltage was applied and removed, and after 3600 s, the optical contrast only attenuated by 0.69%, demonstrating excellent optical bistability.

In recent years, polyimide electrochromic materials have developed rapidly [27,34,35,36,39,40,41,43,44,45,48,49,50,51,52,53,54,55,56]. The performance comparison of PI–ECPs is listed in Table 2. Due to their excellent electrochromic properties, PI–ECPs have been a hot topic of research. Additionally, the further development of devices has made the application of polyimide possible.

### 2.3. Polyamide-Type Electrochromic Polymers (PA–ECPs)

**Figure 5:**
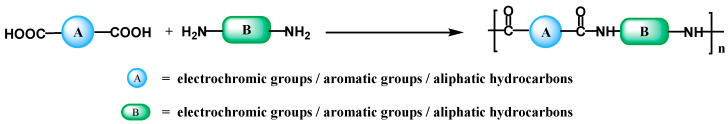
Synthetic routes to the PAs.

The preparation method of polyamide-type electrochromic polymers is relatively simple: the target polymer can be obtained via the condensation of monomers containing dicarboxylic acid groups and aromatic diamines under certain conditions. The general reaction formula is shown in Figure 5. At present, this type of material is the most reported and rapidly developing type of SPECP in related research work.

**Figure 6:**
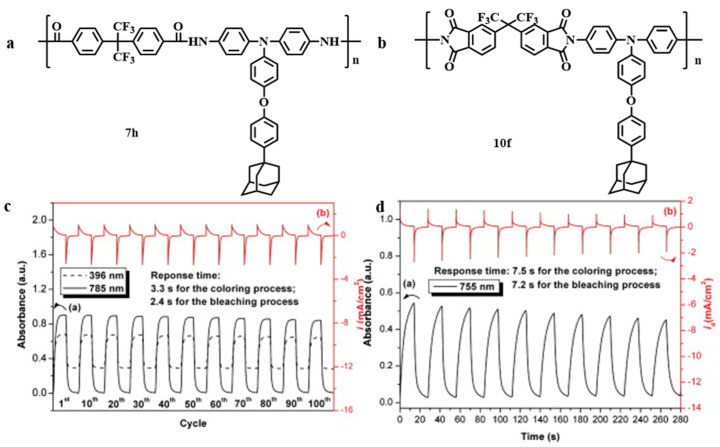
Comparison of stability between PA-7h (**a**) and PI-10f (**b**). The potential step absorptometry and kinetically current consumption of 7h (**c**) and 10f (**d**). Reproduced from ref. [37] with permission.

In 2009, Liou’s group introduced the color-changing units of triphenylamine containing adamantane into the structures of polyamide and polyimide, respectively, resulting in polymers PA–7h and PI–10f (as shown in Figure 6). After conducting electrochromic performance research, it was found that although the types of color changes between the two were similar, the color change potential (1.10 V, vs. Ag/AgCl) of PA–7h was lower than that of PI–10f (1.40 V, vs. Ag/AgCl). In further EC cyclic stability testing, the EC cyclic stability of PA–7h (100 cycles) was also much higher than that of PI–10f (10 cycles). Liou’s group believes that due to the significantly higher electron-withdrawing ability of imides compared to amide structures, the electron cloud density on the triphenylamine structure in PA–7h is higher than that on the triphenylamine structure in PI–10f. Therefore, the electrochemical oxidation potential of PA is relatively low, and the stability of the formed free radical cations is relatively higher [37,57].

Based on the PA–ECPs system, Hsiao’s group systematically studied the effects of groups with different push–pull electron abilities on the performance of electrochromic polymers containing triphenylamine in subsequent works. It was found that the introduction of electron-donating groups such as piperidine [58] and morpholine [59] in the triphenylamine unit structure can significantly decrease the onset potential of the color change of PA–ECP, thereby enhancing the stability of the EC cycle. On the contrary, if an electron-withdrawing group such as cyano [60], trifluoromethyl [61], or methylsulfonyl [62] is introduced into the same triphenylamine structural unit, the color change initiation potential of the corresponding PA–ECP is significantly increased, thereby reducing the EC color change cycle stability of the material. However, the introduction of these electron-withdrawing groups also leads to better optical transparency and higher contrast to the PA–ECPS color-changing film [63]. Similarly, by changing the push–pull electron ability of these groups, the effective regulation of the bandgap of such PA–ECPs can be achieved, and thus, effective regulation of the material’s absorption spectrum and color can be achieved.

**Scheme 3:**
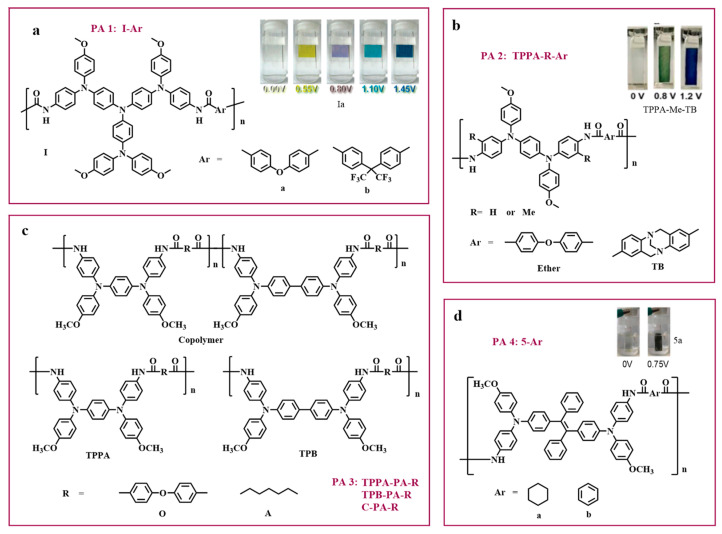
The polymer structure of (**a**) PA 1:Ia, Ib (**b**) TPPA (**c**) TPPA, TPBPA and Copolymer of both and (**d**) PA 4: 5a, 5b.

Liou’s group, for the first time, combined the starburst triphenylamine derivative structure containing multi-EC active N centers with amide structure; they successfully synthesized PA 1 series polymers PA 1–la and PA 1–lb (Scheme 3a), which can realize many color changes in the near-infrared region [64]. Due to the large, conjugated structure and multiple methoxy electron-donating groups of the starburst triphenylamine derivative units, the PA 1 series of polymers exhibit electrochemical characteristics of low initial potential, good electrochemical cycle stability and stable free radical cation during electrochemical oxidation and reduction. During the electrochromic process, the PA 1–Ia film undergoes three highly reversible oxidation processes and one irreversible oxidation process, accompanied the color change from colorless (Y: 90; x, 0.313; y, 0.331) to yellow (Y: 50; x, 0.398; y, 0.461), purple (Y: 21; x, 0.278; y, 0.271), blue-green (Y: 20; x, 0.252; y, 0.315) and dark blue (Y: 7; x, 0.208; y, 0.229). At the same time, the polymer exhibits considerable cycling stability and coloring efficiency. Driven by a voltage of 0.55 V (vs. Ag/AgCl), the PA 1–Ia thin film exhibits high coloring efficiency in the near-infrared region (CE = 290 cm^2^/C at 1170 nm) and reversible electrochromic stability (3.80% decay of color efficiency after 10,000 cycles). When the driving voltage increased to 0.8 V (vs. Ag/AgCl), the CE of the polymer film increased to 339 cm^2^/C at 1100 nm, and after 10,000 cycles, the CE only decreased by 6.78%.

Contrast is one of the important performance indicators of electrochromic polymer materials. An ideal electrochromic material should maintain high transparency under normal conditions while keeping low optical transmittance under working conditions. Among them, the color change from colorless to black holds an important position [65]. So far, there are still very few polymer materials that can achieve color changes from colorless to black. They should have the characteristics of total reflection of light in the colorless state and full absorption of the visible spectrum in the black state [21]. Most of the black electrochromic materials are designed based on the color mixing theory; that is, the polymer is introduced with chromophore groups with different absorption wavelengths, and the material absorbs the visible spectrum by adjusting the proportion of different chromophore groups, thereby achieving the black display effect [66,67,68,69]. However, such designs typically result in polymers having a higher operating voltage, which is an important factor leading to polymer instability.

In 2017, Liou’s group designed and manufactured a bipolar system based on electrochromic polyamides (PAs) derived from tetraphenyl-phenylenediamine (TPA), N,N,N’,N’-tetraphenyl-phenylphenylenediamine (TPPA), tetraphenylbenzidine (TPB), and heptyl viologen (HV). Among them, the first oxidation stage of TPPAPA (Scheme 3c) is green (with visible absorption peaks of about 430 and 600 nm), the first oxidation stage of TPBPA (Scheme 3c) is red (peak of about 486 nm), and the first reduction stage of HV is blue (wide band, center about 603 nm). The absorption range of this system almost completely covers the entire visible spectrum, indicating a black state externally [70]. The author prepared electrochromic films on ITO glass by blending PA of TPPAPA and TPBPA, and PA obtained via the copolymerization of TPPA and TPB, respectively, while HV was introduced into electrolyte gel to prepare electrochromic devices (ECDs). The ECDs prepared via the two methods exhibit similar electrochromic properties. HV is a cathode electrochromic material that not only absorbs blue light but also effectively captures charges by integrating it into the gel electrolyte. This increases the rate of charge exchange and lowers the polymer’s working voltage, which improves the system’s overall performance. Furthermore, by increasing the thickness of the polymer film to 1 μm and applying a voltage of 0 to 1.9 V, the transmittance of the visible region and the NIR of the ECD is effectively reduced to only 1%, thus realizing true all black (L*: 6.5; a*: −3.5; b*: 4.9). However, in the neutral state, the transmittance of the visible spectrum only decreases to 55%, that is, the color in the fading state is not ideal.

In 2020, Guan’s groups combined triphenylamine with tetraphenylethylene to form a novel color-changing unit with a large conjugated structure and successfully prepared PA 4–5a (as shown in Scheme 3d) that can change from colorless (L*: 95, a*: −4, b*: 9) to black (L*: 13, a*: 2, b*: 11) [71]. In addition, the author introduced electron-donating methoxy groups in the para-position of the TPA amino group, which reduces the oxidation potential and enhances the electrochemical stability of the polymer. Compared to the high driving voltage (greater than 1.5 V) of most electrochromic materials that can be displayed as black, the driving voltage of PA 4–5a can be as low as 0.75 V (vs. Ag/AgNO_3_). Meanwhile, PA 4–5a has excellent electrochromic performance. It has an optical contrast of 87.9%, a coloring efficiency of 260 cm^2^/C, and a coloring/fading time of 2.04/1.45 s.

The thickness variation of EC thin films will have a significant impact on parameters such as transparency, color change potential, coloring efficiency and response time. Although a thicker EC film has a greater discoloration effect (higher coloring efficiency), it can also cause issues like increased discoloration potential, a slower reaction time, reduced transparency in the neutral state, and even a major impact on the stability of the EC discoloration cycle.

Liou’s group has carried out a lot of research efforts to address these issues [72,73,74,75]. One technique is to build electrolyte salts into an EC thin film, which is subsequently cleaned to get rid of the salts. The electrolyte salts that are so removed leave behind pores within and on the exterior of the film that have a specific size. These pores can promote the entry and exit of ions, increase the specific surface area of the EC film, and thus shorten the color change response time of the EC film. However, if the pore size formed by rinsing the electrolyte salt is greater than 2 μm, it is not good for the transparency of EC thin films in a neutral state, and it also reduces the optical contrast during the redox process [72]. The formation of inorganic organic composite electrochromic materials is another effective way to accelerate the electrochemical response. Liou’s group combined metal oxides (TiO_2_ [75], ZrO_2_ [74]) into EC polymers, and the inorganic–organic composite EC materials prepared significantly reduced response time compared to pure EC polymer films. Therefore, it is a direction to develop materials to minimize the size of membrane pores without sacrificing the transmittance [72,73].

In 2022, Liou’s group prepared TPPA-TB and TPPA-Me-TB by adding Tröger’s base (TB) to triarylamine polyamides (Scheme 3b). Due to the presence of TB, the polymer chain exhibits a unique V-shaped conformation. Therefore, EC thin films based on drop casting of such materials on ITO-coated glass substrates contain intrinsic microporous structures and exhibit high transparency [72]. Under similar membrane thicknesses (350 ± 30 um), it was found that the response time of polymers containing TB was faster than that of polymers without TB. The author believes that this is due to the presence of microporous structures in the films of TB polymers that facilitate ion diffusion. In addition, EC thin films prepared based on TB polymers have excellent electrochromic stability, and the reversibility of transmittance contrast ratio is above 86% after 500 cycles.

In addition, Chen C.’s Group used electrostatic spraying technology for the first time to deposit polyamide materials on ITO glass and obtained thin films with uniform porous structures under the action of electrostatic field forces. At the same time, the introduction of silver nanowires (AgNWs) into the polymer solution does not hinder the formation of porous structures, and the electrochromic response capability can be improved by improving the conductivity of the polymer film [76]. The polyamide material PA–AgNWs achieves a fading/coloring time of 0.8 s/1.2 s for and a coloring efficiency of over 600 cm^2^/C, which is the optimal coloring efficiency for polyamide materials currently.

Polyamide electrochromic materials are the most widely studied materials in SECPs [25,76,77,78,79,80,81,82,83,84,85,86,87,88,89,90,91,92,93,94]. The performance comparison of PA–ECPs is listed in Table 3. Researchers have conducted in-depth research on it, committed to improving its electrochromic performance and promoting the development of electrochromic applications. The application of electrochromic devices based on polyamides will play an important role in intelligent and environmentally friendly societies.

### 2.4. Polyarylketone Type Electrochromic Polymers (PAK–ECPs)

Polyarylketone (PAK) materials are one of the most important components in special engineering plastics. The most representative material among them is polyether ether ketone (PEEK). Compared to PAI, PA, and PI, PAK materials not only have excellent mechanical properties, good thermal stability, corrosion resistance, radiation resistance, and weather resistance [95] but also have better acid and alkali resistance. More importantly, films prepared using such materials often have higher transparency and lighter colors. At present, the reported PAK-type electrochromic polymers (PAK–ECPs) in the literature are mainly divided into polyarylether ketone type ECPs (PAEK-ECPs) and polyarylamine ketone type ECPs (PAAK ECPs). Like polyamide and polyimide, PAK–ECPs have a wide range of potential applications in related domains and can be developed by generating corresponding groups to construct D-A structures.

**Scheme 4:**
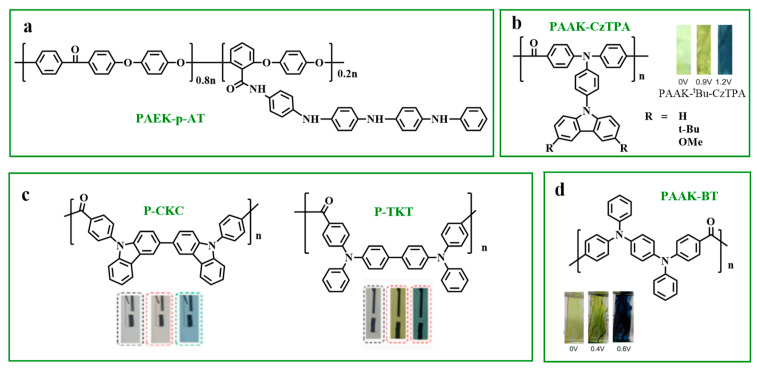
The polymer structure of (**a**) PAEK–p–AT (**b**) PAAK–CzYPAs (**c**) P–CKC, P–TKT and (**d**) PAAK–BT.

The research on PAEK–ECPs was conducted relatively early. In 2012, Chao’s group reported a poly (aryl ether ketone) electrochromic polymer (PAEK–p–AT) (Scheme 4a) containing oligoaniline (n = 3) segments in the side group [96]. This polymer is obtained via the copolymerization of difluoro benzophenone, difluoro oligoaniline monomers, and diphenol in a molar ratio of 0.8:0.2:1.0. Due to the presence of 20% oligoaniline side chain structure, the compactness and regularity between polymer chains can be reduced. So, even if 80% of the polyether ether ketone copolymer segment structure exists, the polymer can still exhibit excellent solubility in various polar solvents such as THF, NMP, DMAc, and DMSO. This facilitates the preparation of variable EC films using its solution. The research results indicate that the electrochromic performance of PAEK–p–AT was relatively outstanding at that time. During the first oxidation, the coloring efficiency of PAEK–p–AT reached 75.9 cm^2^/C at 700 nm. In addition, during the process of changing from gray to dark blue, the optical contrast of the film is 30%, and the coloring and fading times are 4.2 s and 2.0 s, respectively.

**Figure 7:**
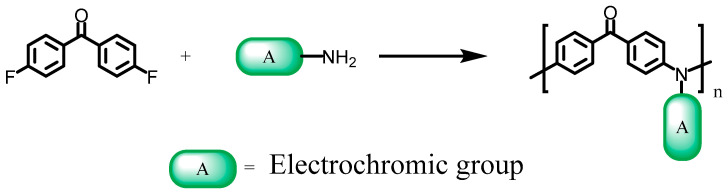
Synthetic routes to the PAKs.

In 2011, according to the reaction mechanism of nucleophilic polycondensation, Chen successfully realized the polymerization of aniline monomer and difluorophenone for the first time under the condition of potassium carbonate as a catalyst. He prepared a type of high thermal stability (T_g_&TGA) polymer PAK–Cz with a main chain containing triphenylamine structural units and a side chain containing carbazole structural units, which can easily achieve kilogram scale quantitative production. (Figure 7) Chen named this polymerization method a two-step C-N coupling reaction [97]. This synthesis method utilizes inorganic alkaline catalysts, which are cost-effective and easy to remove, resulting in reduced environmental pollution. In addition, the reactants for carbon-nitrogen coupling are aniline monomers. Aniline is an important monomer for synthesizing SPECPs due to its rich variety, low cost, and simple and easily obtainable structure. However, it was not until 2018 that Chen’s group applied PAK–Cz materials and their polymerization methods to the research work in the field of electrochromism.

Chen’s group designed and synthesized a series of nonconjugated poly (arylaminoketone) (PAAK) based electrochromic materials with carbazole-9-yltriphenylamine (CzTPA) units, as shown in Scheme 4b [98]. These PAAK–ECPs also exhibit excellent thermal performance: they do not show significant weight loss at 500 °C, and their glass transition temperature ranges from 200 to 256 °C. At the same time, they are colorless under normal conditions and turn yellow (first oxidation stage) and blue (secondary oxidation stage) after applying voltages of 0.9 V and 1.2 V, respectively. The PEEK–CzTPA–*^t^*Bu film has a coloring time of 5.6 s and a fading time of 0.6 s at 730 nm in the process of changing from colorless to blue and has an optical contrast of up to 87%, showing excellent electrochromic performance.

In 2023, Chen’s group constructed electrochromic polymers P–CKC and P–TKT on the surface of ITO substrate through electrochemical polymerization, with structures shown in Scheme 4c [99]. Compared with other film-forming methods, electrochemical deposition has the characteristics of simple operation, low environmental requirements, and can be carried out at room temperature and pressure [100]. Moreover, the polymerization reaction and film formation process can be carried out simultaneously, resulting in extremely high preparation efficiency. Therefore, the method of electrochemical deposition has great value. This method results in a thinner EC color change layer and better film quality. Among them, polymer P–TKT has a coloring efficiency of 498.2 cm^2^/C at 713 nm, with coloring and fading times of 2.0 s and 1.0 s. After 1000 s of cycling, its contrast change (difference in transmittance between coloring and fading states) remains at 42.5% of the initial (first cycling), demonstrating excellent electrochromic performance.

In order to achieve the application purpose of low-cost and quantitative production of PAK–ECPs, in 2023, Chen’s group prepared P–TKT EC polymers with the same structure through chemical synthesis, which have the advantages of low-cost and quantifiable preparation. In addition, the author also prepared PAAK–TT EC materials with a biphenyl structure, as shown in Scheme 4d [101]. Both P–TKT and PAAK–TT EC thin films can achieve reversible color changes from near colorless to green and blue under voltage driving (vs. Ag/AgNO_3_). Among them, the cyclic stability of PAAK–TT material with biphenyl structure is more than 300 cycles, but its optical contrast decreases to 80% of the initial (first cycle). The author believes that this can be attributed to two reasons. On the one hand, the low solubility of such polymers affects the film formation quality of spin coating seriously, and on the other hand, the injection and extraction of charges during the EC cycle disrupt the internal morphology and structure of the EC film and the adhesion between the EC film and the ITO substrate.

Although research on PAEK–ECPs has been conducted in the early stages, there is currently a limited number of studies and reports available. However, the potential application of these materials is readily apparent. In contrast, researchers are more inclined to research and develop polyarylsulfone-type electrochromic polymers.

### 2.5. Polyarylsulfone Type Electrochromic Polymers (PAS–ECPs)

Polyarylsulfone (PAS) is structurally similar to polyarylketones, and it is a thermoplastic polymer with good thermal stability, strong corrosion resistance, and excellent mechanical properties as well [102]. Compared to the ketone carbonyl group in the structure of polyaromatic ketones, polyaromatic sulfone materials often exhibit higher glass transition temperatures. This is because the sulfone group structure limits segment movement, resulting in a larger free volume. On the other hand, the large free volume sulfone group also reduces the extent of close packing between chains. As a result, the melting processing temperature of PAS is lower than that of PAK, which makes it more suitable for further processing applications. Due to the amorphous nature of polyarylsulfone (PAS), both the particles and thin film products of PAS exhibit high transparency. This characteristic fulfills the fundamental criteria for the preparation and development of optical thin films [103,104]. In addition, similar to PAK materials, PAS does not contain amide or imide structures in its structure, so PAS offers significant benefits in terms of acid and alkali corrosion resistance and weather resistance [99]. Based on the above considerations, electrochromic properties of polyarylsulfone can be enhanced by introducing electrochromic groups into its structure. This approach can lead to the structure of a new type of SPECPs with outstanding electrochromic properties. At present, the PAS-type electrochromic polymers (PAS–ECPs) reported in the literature are primarily categorized into two types: polyarylethersulfone (PAES–ECPs) and polyarylamine sulfone (PAAS–ECPs). The color-changing units predominantly employ derivative structures that are based on triphenylamine.

#### 2.5.1. PAES–ECPs

**Scheme 5:**
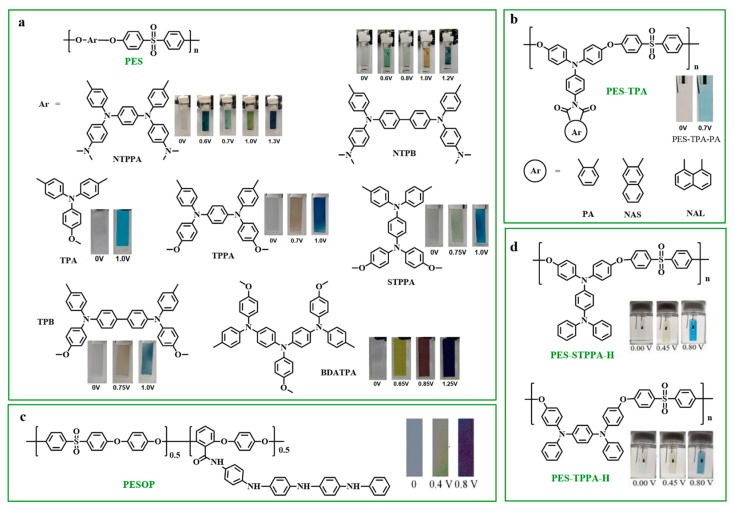
The polymer structure of (**a**) PESs, (**b**) PES–TPAs, (**c**) PESOP, (**d**) PES–STPPA–H, PES–TPPA–H.

In 2018, Liou conducted electrochemical performance tests on a poly (aryl ether sulfone) containing a triphenylamine structure (PES–TPA–H) [105]. It was learned that PES is easily electrochromic crosslinked and easily irreversibly overdoped at high potentials. Therefore, the polymer has poor electrochemical stability and is unsuitable for use as an electrochromic material. Subsequently, Liou’s group further optimized the structure of PES–TPA–H, introducing the electron donating group methoxy (–OMe) and replacing the H atom (Scheme 5a) at the R_1_ position, and polymer PES–TPA–OMe was successfully prepared [106]. The introduction of methoxy groups not only effectively prevents the occurrence of electrochemical crosslinking reactions but also increases the electron cloud density of nitrogen atoms in the triphenylamine structure, which is conducive to stabilizing the cationic free radicals generated during the oxidation process, thereby reducing the electrochemical initial potential of the polymer and improving the electrochemical stability of the material. The research results indicate that PES–TPA–OMe has a certain electrochromic ability, and its EC film can undergo multiple reversible cyclic changes from colorless to cyan when subjected to a driving voltage of 1.0 V (vs. Ag/AgCl). Chen’s group has also researched this issue and proposed solving the problem of the poor electrochemical stability of PES–TPA–H by constructing a D–A structure. A series of PES–TPA polymers, PES–TPA–PA, PES–TPA–NAS, and PES–TPA–NAL, have been developed (Scheme 5b) [107]. They all have good electrochromic ability, especially PES–TPA–PA, which can achieve a reversible change from colorless to blue (52 cycles) at high potentials (1.1 V vs. Ag/AgCl). The coloring/fading time of PES–TPA–NAS film is 4.75/0.84 s, and the coloring efficiency reaches 189.57 cm^2^/C. However, high discoloration potential, a few types of discoloration, and poor EC cycling stability are still prominent issues for PES–TPA–type polymers.

To solve the above problems, TPPA–PES and STPPA–PES were successfully prepared by Liou’s group using a TPPA unit containing bichromic centers instead of a TPA unit [106]. As the applied potential increases from 0.00 V to 0.70 V, the color of TPPA–PES film changes from colorless to light brown and turns blue when the voltage increases to 1.00 V. Under the same conditions, STPPA–PES changes from colorless to green and to sky blue at 1.00 V voltage.

The presence of methoxy groups can prevent coupling reactions between triphenylamine units and also reduce the oxidation potential. TPPA–PES and STPPA–PES exhibit excellent EC performance. However, introducing methoxy groups into a complex structure will inevitably increase the difficulty and production cost of material synthesis. From a practical application point of view, the simpler the structure, the more practical it becomes. Therefore, Chen’s group simplified the methoxy structure in TPPA–PES and STPPA–PES and prepared polymers PES–STPPA and PES–TPPA using fewer synthesis steps, as shown in Scheme 5d [108]. The result showed that there is no side reaction of electrochemical coupling between polymer chains and no significant change in electrochromic performance. Moreover, in the cyclic test of 300 cycles, the optical contrast of PES–TPPA decreased by 17%, while the optical contrast of PES–TPA decreased by 12.6%. Both materials exhibit excellent electrochemical cycling stability during 500 cycles of cycling testing.

Based on TPPA–PES and STPPA–PES polymers, Liou’s group has developed polymer BDATPA–PES (Scheme 5a) with more color-changing characteristics [106]. The EC film based on DATPA-PES can change more colors under voltage driving, transitioning from colorless to yellow-green (0.65 V), purple (0.85 V), and dark blue (1.25 V). This film also demonstrates low color change potential, rapid response speed, and high coloring efficiency. In order to achieve more color changes, Liou’s group developed polymers NTPPA–PES and NTPB–PES (Scheme 5a) in 2020, which contain four color-changing centers [109]. NTPPA–PES displays four color states: blue, light blue, green, and dark blue at voltages of 0.6 V, 0.7 V, 1.0 V, and 1.3 V, respectively. Additionally, after applying voltage, the color of NTPB–PES undergoes several changes: colorless (0 V), green (0.6 V), colorless (0.8 V), orange (1.0 V), and dark blue (1.2 V). Two types of materials were used as anode electrochromic materials, and heptyl viologen (HV) was used as a cathode electrochromic material to prepare electrochromic devices. The spectral changes were consistent with those of the polymer film and showed high coloring contrast.

In addition to the research on the derivative structure of triphenylamine as a color-changing unit, Chao’s Group prepared polyether sulfone materials containing polyaniline groups (PESOP), with the polymer structure shown in Scheme 5c [104]. Under voltage driving, PESOP-based EC thin films can achieve various color changes from gray (0 V) to green (0.4 V) and finally to purple (0.8 V).

#### 2.5.2. PAAS–ECPs

**Scheme 6:**
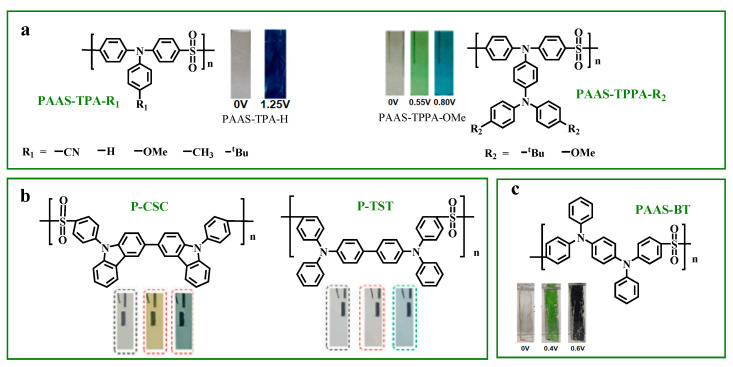
The polymer structure of (**a**) PAAS–TPAs, PAAS–TPPAs (**b**) P–CSC, P–TST and (**c**) PAAS–BT.

Inspired by polyarylamine ketone electrochromic polymers, Chen’s group prepared a variety of polyarylamine sulfone (PAAS) electrochromic polymers using difluorodiphenylsulfone and various aniline structural monomers through a two-step carbon nitrogen coupling method [110]. It is the structural feature that the main chain structure is composed of alternating links between electron-withdrawing sulfone groups and electron-donating triphenylamine units, forming a nonconjugated D-A structure. The general formula is shown in Scheme 6a. All TPA–PAAS can change from colorless or light yellow to blue, with polymer PAAS–TPPA–OMe exhibiting a color change from colorless (normal state) to green (first oxidation state) and then to blue (secondary oxidation state), with an initial potential of 0.64 V. Meanwhile, PAAS–TPPA–OMe exhibits fast response speed (EC process of 6.0/4.3 s), long-term cycle stability (over 3900 cycles), and high coloring efficiency (1131 cm^2^/C) at 928 nm, demonstrating excellent electrochromic performance.

In 2023, Chen’s group prepared polyarylsulfone materials with electrochromic properties more simply by using electrochemical deposition technology, as shown in Scheme 6b [99]. By comparing the electrochromic properties of P–TKT (Scheme 4c) and P–TST materials, the author found that the cyclic stability of polyarylsulfone is weaker than that of polyarylketone materials with similar structures, which is attributed to the more significant structural changes of sulfone-based polymer chains before and after electron loss during the oxidation process, and the greater damage to the integrity of the membrane. Among them, the P–TST material has a coloring time of 2.0 s and a fading time of 1.0 s and has a fast-switching speed. Its coloring efficiency can reach 564 cm^2^/C, which means it has high energy efficiency when used as a device. At the same time, the author explored the reasons for the insufficient cycling stability of the material and believed that ion implantation and extraction were not fully carried out in many repeated cycles. After multiple cycles, the difficult-to-release counter ions accumulate to a certain amount, which eventually leads to the degradation of electrochromic properties.

In view of practical application, PAAS–BT and PAAS–TT (Scheme 6c) with D–A structure were prepared via a simple one-step carbon nitrogen coupling method [101]. Due to the low cost of raw materials (the two aniline monomers were priced at approximately USD 0.43/g and USD 0.15/g, respectively), they can achieve a color change from colorless to yellow and ultimately to blue. Among them, the coloring efficiency of PAAS–BT film reached 160.34 cm^2^/C and maintained 58% of the original optical contrast after 450 cycles. The reason why PAAS exhibits better electrochromic performance compared to PAAK materials with the same donor can be attributed to the stronger electron absorption ability of sulfone groups. It can be produced quantitatively at the kilogram level, as this work is of great significance for the quantitative production and application of electrochromic polymers.

SPECPs have gone through a lengthy process of exploration. Researchers have attempted various structural designs and synthesis methods to prepare electrochromic materials with superior performance. By comparing the development of SPECPs over the years, we can observe significant improvements in their electrochromic performance. Some SPECPs have achieved a switching time of less than 1 s, a coloring efficiency of up to 500 cm^2^/C, an electrochromic cycle stability of 10,000 cycles, and a wide range of color changes. It can be foreseen that SPECPs will have a competitive edge in the electrochromic market in the future.

## 3. Multifunctional Applications of SPECPs

### 3.1. Memory Device

The application of SPECPs to memory devices is the earliest multifunctional application research of this type of material.

**Figure 8:**
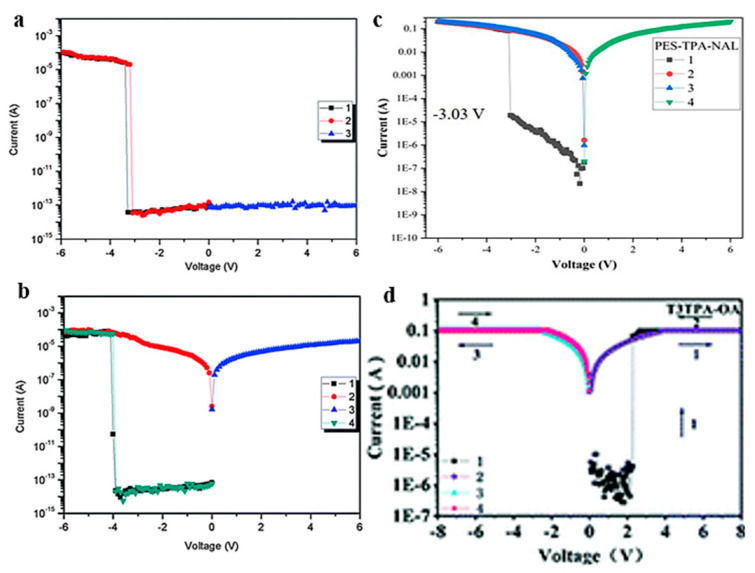
(**a**) Current–voltage (I–V) characteristics of the ITO/**AQ-6FPI**/Al memory device. (**b**) Current–voltage (I–V) characteristics of the ITO/**OAQ-6FPI**/Al memory device. (**c**) Current–voltage (I–V) characteristics of the ITO/PES–TPA–NAL/Al memory device. (**d**) Current–voltage (I–V) curves of T3TPA-OA. Reproduced from refs. [89,107,111] with permission.

In 2012, Liou’s group designed triphenylamine (TPA) polyimide AQ–6FPI and OAQ–6FPI materials with electron-withdrawing hanging anthraquinone groups. In addition to their electrochromic properties, they also demonstrated functionality in memory storage. The device of AQ–6FPI switches to the ON state at a threshold voltage of about −3.5 V and then resets to the OFF state after removing the applied electric field. Without any erasure process, the device can open again at −3.4 V during the second negative sweep, indicating the dynamic random-access memory (DRAM) behavior of AQ–6FPI (Figure 8a). At a threshold voltage of −3.9 V for the first negative sweep, the OAQ–6FPI device instantaneously increases from 10^−14^ to 10^−4^ (high conductivity state), indicating the writing process from the OFF state to the ON state. During the subsequent negative scan and positive scan, the device remains in the ON state, which can be defined as the read process. After turning off the power for 8 min, the memory device returns to the off state without any erasing process, and can be reconnected at a threshold voltage of −3.9 V (line 4); This phenomenon indicates that the memory device has static random access memory (SRAM) characteristics (Figure 8b) [111].

In 2017, Liou’s group tested the information storage function of Vio–PIs and tested the long-term operation of Vio–CH memory devices in their respective ON and OFF states under continuous application of −1 V voltage. After reading the test for 104 s, the current attenuation in the ON and OFF states can be ignored, indicating that the memory device has good stability. In addition, the memory device also has good flexibility and will not crack/deform under severe mechanical bending stress, demonstrating the high feasibility and repeatability of memory behavior [41].

In 2021, Chen’s group tested the memory behavior of ECPs through the current–voltage (I–V) characteristics of ITO/PES–TPAs/Al sandwich devices [107]. For polymer PES–TPA-NAL, the device is in a low conductivity state (OFF state) and then switches to a high conductivity state (ON state) at a threshold voltage of 3.03 V during the first negative scan from 0 to 6.0 V. The on/off current ratio is 10^3^, which can be defined as the “write” process of the storage device. During a negative scan from 0 to 6.0 V (scan 2) or even a positive scan from 0 to 6.0 V (scan 3), the device remains in the ON state as a “read” process. By applying a reverse voltage, the device cannot switch to its original OFF state even after a power outage of about 1 h, indicating that the device has non-volatile storage characteristics (Figure 8c). The threshold voltages of PES–TPA–PA and PES–TPA–NAS are 3.64 V and 3.21 V.

In addition, Niu’s group has also developed the application of electrochromic polyamide materials in memory devices [89,112]. T3TPA monomers were prepared by combining triolefins with triphenylamine, and HBPAs with hyperbranched structure were synthesized, which meets the requirements for preparing memory device materials [89]. The device made of T3TPA–OA material has a low conductivity state between 0 V and 2.26 V, which is equivalent to the signal “0” in inorganic semiconductor memory devices. When the voltage exceeds 2.26 V, the device suddenly transitions from a low conductivity state to a high conductivity state, maintaining a high conductivity state equivalent to signal “1”, which is equivalent to the “write” process of the storage device. In addition, this writing process is irreversible, meaning that the device is a “non-volatile storage device” (Figure 8d).

### 3.2. Supercapacitor

Integrating electrochromic and electrochemical energy storage on one platform can not only intuitively reflect the energy storage state through color changes but also store the energy consumed by color change. In this case, it can be used to drive electronic devices, achieving efficient energy conservation, storage, and utilization [113].

Chen C.’s group synthesized two hyperbranched polyimides (HBPI) with excellent solubility and thermal stability in 2017. Preliminary capacitance tests showed that their specific capacitance value was 69 F/g, which demonstrates their application prospects in electrochromic displays and electrochromic supercapacitors [44].

In 2022, Liou’s group introduced the Tröger’s base (TB) into polyamide materials. Its unique V-shaped conformation allowed the polymer to form a microporous structure, enhancing ion transport in electrochromic films (Scheme 3b). Under the test conditions of 1.0 A/g, the specific capacitance (C_sp_) of TPPA-Ether was 111.8 F/g, while the specific capacitance of TPPA–TB with TB structure increased to 117.5 F/g. Similarly, the C_sp_ of TPPA–Me–TB (165.3 F/g at 1.0 A/g) was higher than that of TPPA–Me–Ether without TB fragments (158.9 F/g at 1.0 A/g). It is worth noting that under high current density, the C_sp_ value of these PA–ECPs films can also be maintained at around 100 F/g. At the same time, these PA–ECP films exhibit significant two-stage color changes, which can evaluate and monitor energy storage from 0.0 V (colorless) to 0.8 V (green) in the first charging state or 1.2 V (blue) in the second charging state [72].

In 2023, Chao’s group prepared a new type of electrically active polyimide containing pentaphenylamine in the main chain and assembled a quasi-solid state electrochromic supercapacitor device (QSES) [114]. The QSES device appears blue at a voltage of 1.94 V, and when the QSES device is discharged to 0 V, its color changes to green. At the same time, the device has excellent photoelectric performance and good cycle stability that after 10,000 charges and discharges at a current density of 0.2 mA/cm^2^, its capacitance retention rate is 85%, and the optical modulation retention rate is 96%. This energy visualization function is conducive to human energy monitoring and management of supercapacitors at any time, once again indicating that QSES devices balance the properties of electrochromism and supercapacitors, and perfectly combine and display the two.

However, there are still difficulties to be solved in integrating electrochromism and capacitors on a single platform. The electrochromic active layer has a requirement for the thickness of the color-changing layer. An excessively thick active layer will result in difficulties in charge transfer and an inability to complete color changes. However, thin active layer materials have limitations on energy storage and cannot provide a large capacitance, resulting in conflicting functions between the two. We believe that improvements in the device may be able to solve this problem. For example, one approach is to increase the contact area of the active layer and fabricate devices with multiple layers of active material. How to find a balance between color changing function and energy storage is a question that researchers need to consider.

### 3.3. Electrofluorochromism

Electrochromic materials are light source-dependent materials that can only be observed with a light source, and the emergence of fluorescence function fills the gap that cannot be observed in the dark of material color change. Fluorescent materials can also emit fluorescence in the dark, and fluorescence quenching occurs after applying voltage. Changes in light and darkness can serve as a warning in the dark. The combination of electrofluorochromism and electrochromism is beneficial for expanding the range of applications for materials and meeting market demand more effectively.

**Scheme 7:**
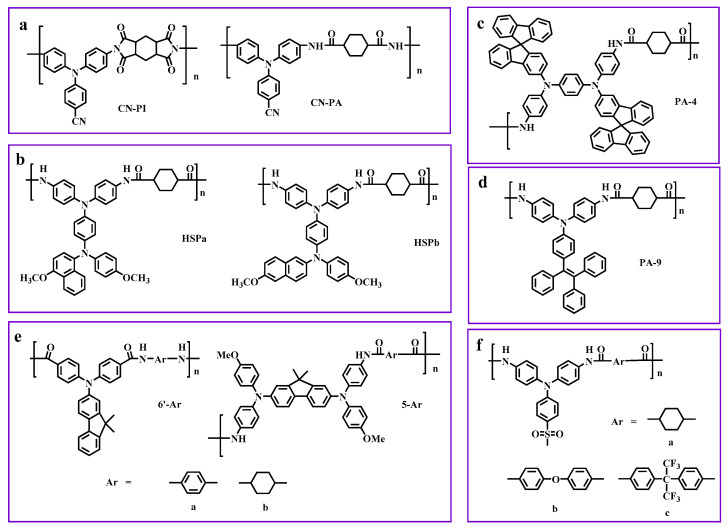
The polymer structure of (**a**) PI–CN, PA–CN, (**b**) HSPs, (**c**) PA–4 (**d**) PA–9, (**e**) 6′–Ar, 5–Ar, (**f**) PA–a, PA–b, PA–c.

As early as 2006, Liou’s group had developed electrochromic polyamide imide with fluorescent properties [34]. Subsequently, in 2013, Liou’s group introduced cyano groups (PI–CN, PA–CN) into polyamides and polyimides with triphenylamine units (Scheme 7a). It is the earliest bifunctional electronic control material in SPECPs that exhibits both EC and EFC characteristics [60].

Chen C.’s group focuses on preparing a series of polyimide and polyamide materials with dual functions of EC and EFC [44,45,62,82,83,84,85,86,87,88,115,116,117,118,119].

In 2019, Professor Chen C.’s research group constructed a new type of electroactive fluorescent group using diphenylamine and pyrene units, which was introduced into polyimide to improve its EC/EFC performance [45]. The obtained polyimide material exhibits a color change from yellow to blue-purple and a fluorescence of yellow-green. Surprisingly, its electrochromic color switching time is 1.7/0.9 s, and the electrofluorochromic switching time is 2.8/0.9 s. This EC/EFC dual functional material can be used to design dual mode color changing/fluorescent displays to achieve high visualization of EC displays in dark or bright environments.

Chen C.’s group introduced fluorescent groups such as TPE (Scheme 7d), tetraphenylethylene, fluorene (Scheme 7e) et al. into polyamides with triphenylamine groups to prepare bifunctional materials with high fluorescence efficiency, contrast, and excellent electrochromic properties [62,82,83,85,86,87,88,116,117,118,119]. Subsequently, in order to improve quantum yield, Chen C.’s group introduced electron-withdrawing sulfonyl groups onto the polymer and constructed a donor–acceptor structure with electron-donating triphenylamine groups, preparing the polymer as shown in Scheme 7f. This polymer not only achieves a fluorescence quantum rate of 32.1% but also exhibits a colorless to purple color change [62]. To further improve the stability and fluorescence efficiency of the material, the author constructed multiple conjugated nitrogen centers (such as bis (diphenylamine)) to increase resonance coupling and introduced helical difluorene (Scheme 7c) with high photoluminescence ability. The designed polymer has a color change from colorless to green and then to blue, as well as a blue-black emission change. Its EC contrast reaches 69.2%, and the number of cycles exceeds 400. The fluorescence quantum yield reaches 44.1%, and the number of cycles of EFC exceeds 800 [88]. In addition, naphthalene has the characteristics of high fluorescence quantum yield, good optical/chemical stability, and easy functionalization. The author introduced it into polyamide to improve the electrical response speed and cyclic stability of the material (Scheme 7b). At the same time, the influence of different positions of naphthalene substitution on the coplanarity and charge transfer intensity of diphenylamine naphthalene was also explored, which in turn affects the glass transition temperature, color efficiency, fluorescence quantum efficiency, fluorescence emission wavelength, and response speed [87].

### 3.4. Infrared Stealth

The principle of infrared stealth function is that electrochromic materials absorb infrared light at different voltages and adjust the reflectivity or emissivity of infrared devices to be consistent with the surrounding environment, achieving the purpose of stealth. On the other hand, electrochromic materials can control the surface temperature of the target by adjusting the reflectivity of mid to far infrared light, better hiding the target [120]. This issue has been studied by many researchers [53,64,110,121]. In 2020, the PAAS–TPPA–OMe designed by Chen’s group showed a wider absorption band at 928 nm after applying a positive voltage of 0.55 V. In 2023, Niu’s group introduced a fluorene group into polyimide. Additionally, at a voltage of 0.7 V, the strong intramolecular electron coupling generated by the fluorene group bridging resulted in a wider near-infrared absorption peak of polyimide at 1254 nm, demonstrating great potential in near-infrared stealth applications.

### 3.5. Other Functions

In 2008, Liou’s group conducted gas separation tests on PI–SPECPs and studied the transport characteristics of these polyimides for CO_2_, CH_4_, O_2_, and N_2_ [35]. The oxygen permeability coefficient (Po_2_) of IIb containing 4-methoxy substitution is 4.28 barriers, which is higher than that of Ib without 4-methoxy substitution (Po_2_ = 0.69). The results indicate that introducing larger TPA units into aromatic polyimides can improve gas transport performance by increasing permeability while its permeability selectivity only slightly decreases. In 2011, the polyimide I-6F prepared by Liou’s group had high permeability and CO_2_/CH_4_ separation selectivity, which was higher than the Robeson upper limit, and the gas permeability of CO_2_ can reach 229 [47].

The multifunctional applications of electrochromic materials are also being utilized in many aspects. Niu’s group has carried out a lot of research on this [46,53,122,123,124,125,126]. In 2019, Niu’s group developed three polyimide materials (DPA–NIME–PI, TBPA–NIME–PI, and Cz–NIME–PI) that can respond to four stimuli: light, pH, explosives, and voltage. These materials introduce triarylamine (TAA) and naphthalimide (NI) groups onto polyimide, resulting in four functions: photodetectors, electrochromic, fluorescent detectors, and storage devices [125]. The TAA group provides the electrochromic performance and resistance storage function of polyimide, while the NI group provides the fluorescence response function to pH, metal ions and explosives. These three materials all exhibit yellow-green fluorescence, with fluorescence quantum yields ranging from 28.5% to 47.9%. Their fluorescence intensity decreases with increasing pH, and they exhibit a strong linear relationship, making them excellent sensors for measuring pH concentration sensors. In addition, trace concentrations of explosives such as TNT and TNP can cause fluorescence quenching of the material, thereby achieving a sensitive explosive detection effect. The resistance of these materials can also change abruptly with the change of current, thus achieving information storage. Therefore, they can be used as active layer materials for memory devices.

In 2023, Niu’s group synthesized polyimide with fluorene group, which has good electrochromic properties and high fluorescence efficiency (48%), and good conduction function in detecting TNP explosives [53]. Due to the presence of electron-withdrawing nitro groups in TNP, photoinduced electron transfer (PET) and resonance energy transfer (FRET) can occur between TNP and polymers, leading to fluorescence quenching. As the concentration of TNP increases, the fluorescence intensity of polyimide P1 gradually decreases. When the concentration of TNP is in the range of 0–10 μg/mL, the PL quenching of the polymer exhibits a linear relationship with the equation, with a correlation coefficient of 0.989. That is to say, within a certain range, the concentration of TNP in explosives can be quantitatively detected based on the fluorescence intensity of P1.

In the same year, Niu‘s group introduced benzophenothiazine (BPT) into polyamide materials, and the prepared polyamide (TPA-–12–BPT) had the function of pH detection [126]. TPA–12–BPT solution can change from colorless to blue in both organic and inorganic acid environments. Subsequently, an alkaline environment was provided, and the solution returned to colorless. This reversible acid-base color change is expected to enable the material to be applied in solid-state detectors for acidic gases.

The combination of electrochromic and multifunctional properties makes these new polyimides a new type of processable special engineering plastic-based electrochromic polymer.

## 4. Challenges and Recommendations

Despite having a relatively short development history of just over a decade, special engineering plastics-based electrochromic polymers have become an important area of research in the field of electrochromic materials. In terms of electrochromic performance, SPECPs have a prominent feature of undergoing reversible changes from colorless to other colors. This feature forms a perfect complementary relationship with conjugated polymer electrochromic materials, which can change from colored to colorless. As a result, electrochromic materials can effectively meet the changing color needs in more application scenarios. In addition, due to their excellent properties of high thermal stability, good mechanical properties, and strong weather resistance, the SPECPs have a wider range of applications compared to other electrochromic polymers, particularly under extremely harsh conditions, which will have greater application advantages. In addition, benefiting from the simple synthesis methods and ease of solvent processing, they have significant application advantages in achieving quantitative production at lower costs and being suitable for roll-to-roll processing. However, there are still some problems and difficulties that need to be urgently addressed in SPECPs.

(1) The electrochromic cycle stability is poor, and there is still a significant gap compared to EC-conjugated polymers. Since SPECPs are nonconjugated polymers, they are less conductive than EC-conjugated polymers, which results in a much higher color change voltage than EC-conjugated polymers. High discoloration potential can easily trigger electrochemical side reactions, which in turn lead to a significant decrease in EC cycle stability. In addition, the inter-chain stacking of SPECPs with a non-conjugated structure has poor regularity and relatively weak inter-chain acting force, so the color-changing film is prone to be damaged and deformed due to repeated mechanical stress action during repeated electrochemical de-doping. The stability of EC circulation is greatly reduced. How to improve the high conductivity of SPECPS and enhance its link force will be the key to solving this problem.

(2) The structure of color-changing units tends to become more complex, resulting in long preparation cycles and high production costs. Triphenylamine-derived structures are still the main structural units in EC materials currently. In order to pursue higher EC performance, researchers have developed monomers derived from triphenylamine, which have more color-changing types and lower starting potential for color change (as pre-polymerization monomers for subsequent polymerization steps). Such monomers have a significant effect on improving the EC performance of SPECPs. However, due to their complex structural characteristics, SPECPs often face common problems such as complex synthesis steps, high raw material costs, and low production efficiency, which greatly weakens the production and application advantages of SPECPs. How to design and develop new EC prepolymer monomers with simple synthesis methods and excellent EC performance is another challenge facing researchers. By summarizing the experiences of predecessors, a well-designed D-A structure can enhance the performance of electrochromism while simplifying the structural design. On the other hand, the introduction of theoretical calculations can guide the design and synthesis of SPECPs. By simulating the distribution of electronic energy levels in polymer molecules, theoretical calculations can calculate the energy gap, predict the absorption spectrum, minimize trial and error in color design, and provide guidance for molecular design [127,128].

(3) Lack of in-depth research on the construction of EC devices based on SPECPs. Due to the short development time of SPECPs, research on the design and synthesis of new types of SPECPs constitutes the main focus in this field. However, there are few reports on targeted research on how to construct efficient EC devices based on the corresponding materials. In terms of device construction, researchers typically utilize the device construction method and device structure of EC-conjugated polymers to prepare corresponding SPECP EC devices. In most cases, such EC devices cannot fully demonstrate the excellent characteristics of SPECPs. This is because researchers have overlooked the significant performance differences between non-conjugated EC polymers and conjugated EC polymers. The excellent EC characteristics of SPECPs can be fully exploited by studying the structure of the components, component components, and processing methods. This research aims to explore the optimal processing technology and structure design for EC materials with different characteristics. Only in this way can we further promote the rapid development of SPECPs. From a structural standpoint, there is still a need to explore assembly methods for transmission devices, reflection devices, and integrated electrochromic devices based on SPECPs. In addition, researchers also need to design and further develop new device structures. From the perspective of device components, the selection of electrodes needs to meet two basic performance requirements. First, the working voltage range needs to be consistent to prevent electrode failure caused by overvoltage. Second, there should be a balance in the charge between the color-changing layer and the counter electrode. This means that during the working process, the number of injected and extracted charges from both sides should be roughly equal. The electrolyte layer also needs to meet conditions such as high ion conductivity, transparency, chemical stability, and electrochemical stability. To meet these conditions, researchers need to thoroughly screen and match the various components of the device. In terms of processing methods, when applying 3D printing, spraying, screen printing, and other techniques to SPECPs, it is necessary to further explore their processing conditions. Therefore, the construction of devices poses a barrier that current SPECPs must overcome in order to further develop.

## 5. Conclusions

In this paper, we introduce and summarize the recent development of special engineering plastic-based electrochromic polymers. Based on their excellent electrochromic performance, mechanical properties, and thermal stability, SPECPs have a wide range of applications in transmission displays, wearable electronic products, electronic skins, electronic paper, and visual energy storage. They are also expected to rapidly develop in fields such as flexible fabrics, sensors, information encryption, and mobile phones. We also discussed the multifunctional applications of SPECPs in this article, and further optimization of the device preparation technology will accelerate the commercialization process of SPECP applications. Even though the current performance of SPECPs falls short of the demands of market-oriented applications and there are pressing problems that researchers must address, we believe that these materials have significant development potential and will eventually be used in everyday applications.

## Figures and Tables

**Table 1:** Electrochromical performance of PAIs.

S/N	PAI– ECPs	T_g_ (°C)	T_d_ at 5% Weight Loss (°C)		Original Color	Oxidation Potential and Color (V) ^a^				Reduction Potential (V)		E_onset_ Ox (V) ^b^	E_g_/eV ^c^	ΔT%	Tc/Tb (s) ^d^	Cycle	CE (cm^2^/C) ^e^	REF
			N_2_	Air		1st E^1/2^		2nd E^1/2^		1st E^1/2^	2nd E^1/2^							
**1**	PAI–2M	279	495	483	brownish	0.67 (green)		1.08 (deep blue)		-	-	0.52	3.13	0.202	3/2	100 cycles	305	[28]
**2**	2a	-	-	-	pale brownish	0.65 (green)		1.02 (blue)		-	-	-	2.95	-	4/3	10 cycles	-	[29]
**3**	4c	-	-	-	colorless	0.69 (yellow)		1.04 (blue)		-	-	0.42	3.09	-	-	-	-	[30]
	4f	-	-	-	pale yellow	0.69 (yellowish green)	0.89 (green)		1.05 (blue)	-	-	0.47	2.89	-	-	-	-	
	4g	-	-	-	pale yellow	0.63 (deeper yellow)		0.69 (yellowish green)		-	-	0.42	2.70	-	-	-	-	
**4**	5c	278	533 (10%)	540 (10%)	nearly colorless	0.93 (pale blue)		-		−1.10	-	0.82	3.06	39%	8.5/4.2	100 cycles	-	[31]
**5**	1	340	635 (10%)	632 (10%)	pale yellow	1.08 (purplish blue)		-		−1.18 (pink)	−2.01 (red-brown)	0.90	2.68	-	3.1/1.3	10 cycles	-	[32]
	2	352	650 (10%)	621 (10%)	yellow	0.81 (deep blue)		1.00		−1.19	−2.11	0.65	2.15	-	-	30 cycles	-	
	3	335	623 (10%)	626 (10%)	yellow	0.86 (bluish gray)		-		−1.15 (orange)	−2.04 (red-brown)	0.66	2.29	-	3.5/1.3	10 cycles	-	
**6**	1a	-	-	-	colorless	1.3 (black)						1.0	3.1	91.4%	1.3/1.1	5000 cycles	99	[33]
	2a	-	-	-	colorless	1.2 (blue)						0.9	3.2	81.2%	2.2/3.7	5000 cycles	78	

^a^ Versus Ag/AgCl. E^1/2^: Oxidation half-wave potentials from cyclic voltammograms. ^b^ Oxidation initiation potential. ^c^ Calculated from the polymer films using the equation: Eg = 1240/λonset (energy gap between HOMO and LUMO). ^d^ T_c_: Timescale at oxidation state reaching 90% of the full switch in transmittance. T_b_: Timescale at reduction state reaching 90% of the full switch in transmittance. ^e^ Coloration efficiency is derived from the equation: η = ΔOD/Q while Q is Ejected charge, determined from the in situ experiments.

**Table 2:** Electrochromical performance of PIs.

S/N	PA–ECPs	Tg (°C) ^a^	Td at 5% Weight Loss (°C) ^b^		Original Color	Oxidation (V) and Color ^a^					Reduction Potential (V)				E_onset_ Ox (V) ^b^	E_g_/eV ^c^	ΔT%	Tc/Tb (s) ^d^	Cycle	CE (cm^2^/C) ^e^	REF
			N_2_	Air		1st E^1/2^		2nd E^1/2^			1st E^1/2^		2nd E^1/2^								
**1**	DH–PI	-	-	-	colorless	0.36 (black)		-			-		-		0.31	3.14	-	2.1/1.7	500 cycles	212	[27]
	TH–PI	-	-	-	pale yellow	0.54 (light green)		0.15 (black)			-		-		0.40	2.88	-	2.5/1.5	230 cycles	177	
	TOH–PI	-	-	-	colorless	0.42 (blue)		0.93 (dark purple)			-		-		0.25	3.23	-	10.7/6.8	500 cycles	113	
**2**	F	310	604	553	pale yellow	1.10 (green)		1.44 (dark blue)			-		-		-	3.29	-	3/1.5	10 cycles		[34]
**3**	PI–VIa	292	475	490	colorless	0.65 (green)		1.00 (blue)			-		-		0.56	2.12	60% (1st) 84% (2nd)	3.26/1.47 (1st) 4.22/1.42 (2nd)	30 cycles	118 (1st) 123 (2nd)	[35]
**4**	6e	305	538 (10%)	519 (10%)	colorless	0.73 (green)		1.06 (blue)			-		-		0.60	2.90	44% (1st) 98% (2nd)	2.4/2.4 (1st) 3.0/2.4 (2nd)	50 cycles	214	[36]
**5**	6d	290	497	514	pale yellowish	0.63 (cyan)		0.95 (bluish purple)			-		-		0.53	2.99	-	16/23	100 cycles	180	[39]
**6**	TPA–PTPI	-	-	-	Indian red	0.99 (deep purple)		-			−0.67 (bright violet)		-		-	-	73% (Ox) 60% (Red)	6.1/4.2 (Ox) 2.9/1.3 (Red)	100 cycles	371 (Ox) 271 (Red)	[40]
**7**	Vio–PM	170	250	245	pale yellow	1.02 (dark cyan)		-			−0.55 (light purple)	−0.76 (dark purple)	−0.95 (light green)	−1.31 (magenta)	0.85	2.99	64%	-	-	-	[41]
	Vio–BP	175	255	255	pale yellow	0.96 (dark cyan)		-			−0.32 (purple)	−0.84 (dark yellow)	−1.21 (dark olive green)	−1.38 (navy)	0.79	3.12	50%	-	-	-	
**8**	9Ph–PMPI	265	460	460	colorless	0.60 (yellow)	0.90 (purple)	1.25 (blue)		1.50 (bluish-green)	−0.9		−1.5		0.35	2.13	82% (2nd) 80% (4th)	3.99/0.90 (Ox 1st) 3.12/1.74 (Ox 2nd)	1000 cycles	226 (1st) 280 (2nd)	[43]
**9**	6FDA–HBPI	286	-	-	near colorless	0.46 (yellow)	0.72 (red)	0.99 (indigo)		1.24 (black)	-		-		0.33	2.86	63% (1st) 42% (2nd) 44% (3rd) 49% (4th)	2.6/1.9 (1st) 1.8/4.3 (2nd) 2.3/1.8 (3rd) 4.9/3.1 (4th)	10,000 s	196 (1st) 92 (2nd) 86 (3rd) 89 (4th)	[44]
**10**	PI	-	-	-	yellow	0.79 (blueviolet)		-			-		-		0.36	-	-	1.7/0.9		155	[45]
**11**	PI–4	221	434	-	colorless	0.99 (light orange)		1.45 (blue)			-		-		0.80	1.84	28%	1.6/1.2	2000 cycles	186	[46]
**12**	1a	339	425	390	pale green	0.42 (light green)		0.70 (light bluish green)			−0.98		-		0.28	1.69	57%(3rd)	-	-	-	[48]
	2a	322	520	510	pale yellow	0.48 (green)		0.78 (dark blue)			−1.05		-		0.34	2.20	81% (1st) 76% (2nd)	-	-	-	
	3a	270	440	430	pale yellowish	0.52 (yellowish-green)		0.74 (purple)			-		-		0.40	-	66% (1st) 77% (2nd)	-	100 cycles	-	
**13**	I–DS	340	470	460	pale yellow	1.24 (green)		-			−0.82		-		1.07	2.35	77%	-	10 cycles	136	[49]
**14**	P3	312	-	-	colorless	0.75 (deep purple)		-			-		-		-	-	62%	6.5/5.1	500 cycles	193	[50]
**15**	PAA–IM–1	-	-	-	light yellow	1.26 (blue)		-			-		-		-	3.22	-	1.7/0.8	50 cycles	834	[51]
	PAA–IM–2	-	-	-	light yellow	1.35 (green)		-			-		-		-	3.16	-	3.8/1.6	50 cycles	523	
**16**	PyTPMPI	-	317	-	colorless	0.90 (light blue)		-			-		-		0.89	3.08	-	3.69/7.08	1000 s	72.73	[52]
	PyTPTPI	-	299	-	yellow	0.90 (dark blue)		-			-		-		0.91	3.12	-	3.25/5.78	1000 s	190.91	
	PyHPMPI	-	348	-	colorless	0.98 (light blue)		-			-		-		082	3.21	-	3.13/3.00	1000 s	72.42	
	PyHPTPI	-	296	-	yellow	0.95 (dark blue)		-			-		-		0.85	3.10	-	1.19/3.30	1000 s	42.30	
**17**	PI–6A	-	354	-	light yellow	0.82 (brown yellow)		1.07 (deep yellow)			-		-		0.59	3.33	55%	10.0/7.7	600 cycles	191	[53]
**18**	CzpP–SPP	-	422	-	colorless	0.7 (green)		1.2 (black)			-		-		-	-	50%	2.8/2.9	2000 cycles	108	[54]
	CzpP–PREP	-	410	-	colorless	0.93 (yellow)	1.19 (yellow−green)		1.38 (green−black)		-		-		-	-	30%	3.3/2.5	2000 cycles	90	
	CzpP–FRNP	-	454	-	colorless	0.91 (yellow)		1.33			-		-		-	-	34%	3.6/2.7	2000 cycles	187	
**19**	P1	-	411	-	yellow	0.69 (reddish brown)		0.82 (inky blue)			-		-		0.41	3.74	26%	3.2/8.7	-	239	[55]
	P2	-	405	-	yellow	0.95 (reddish brown)		1.15 (inky blue)			-		-		0.51	3.43	52%	3.1/2.4	200 cycles	310	
	P3	-	378	-	yellow	0.67 (reddish brown)		0.84 (inky blue)			-		-		0.31	3.32	34%	2.3/2.8	300 cycles	170	
**20**	P1	307	-	-	yellow-orange	0.91 (blue)		-			−0.94		−1.56		0.72	1.46	75%	1.98/5.14	100 cycles	316	[56]
	P2	280	-	-	pale yellow	0.90 (light blue)		-			−1.41		-		0.71	1.82	24%	17.92/18.14	100 cycles	158	
	P3	325	-	-	pale yellow	0.92 (light blue)		-			−1.43		-		0.71	1.77	19%	17.22/18.39	100 cycles	108	

^a^ Versus Ag/AgCl. E^1/2^: Oxidation half-wave potentials from cyclic voltammograms. ^b^ Oxidation initiation potential. ^c^ Calculated from the polymer films using the equation: E_g_ = 1240/λ_onset_ (energy gap between HOMO and LUMO) ^d^ T_c_: Timescale at oxidation state reaching 90% of the full switch in transmittance. T_b_: Timescale at reduction state reaching 90% of the full switch in transmittance. ^e^ Coloration efficiency is derived from the equation: η = ΔOD/Q while Q is Ejected charge, determined from the in situ experiments.

**Table 3:** Electrochromical performance of PAs.

S/N	PA–ECPs	Tg (°C)	Td at 5% Weight Loss (°C)		Original Color	Oxidation (V) and Color ^a^		Reduction Potential (V)		E_onset_ Ox (V) ^b^	E_g_/eV ^c^	ΔT%	Tc/Tb (s) ^d^	Cycle	CE (cm^2^/C) ^e^	REF
			N_2_	Air		1st E^1/2^	2nd E^1/2^	1st E^1/2^	2nd E^1/2^							
**1**	BCT–1	-	-	303	pale yellow	0.67 (green)	-	-	-	-	-	4.25%	3.71/3.89	-	266	[25]
**2**	PA–AgNWs-ES	-	-	-	-	-	-	-	-			86.9%	0.8/1.2	-	697.1	[77]
**3**	6g	248	495	490	colorless	0.48 (green)	0,84 (blue)	-	-	0.36	2.95	86% (2nd)	3.04/1.87 (1st) 4.02/2.02 (2nd)	1000 cycles	285 (1st) 272 (2nd)	[78]
**4**	Ib	262	505	490	colorless	0.58 (green)	0.96 (blue)	-	-	0.48	2.94	87% (2nd)	4.21/1.17 (1st) 3.96/2.04 (2nd)	1600 cycles	276 (1st) 174 (2nd)	[79]
**5**	4g	312	574 (10%)	576 (10%)	pale yellow	1.0 (greenish-gray)	1.5 (dark purplish-gray)	−1.90 (bright yellow)	−2.4 (reddish orange)	-	2.74	-	3.8/1.6	100 cycles	112	[80]
**6**	5b	248	465	442	colorless	0.75 (pale green)	0.92 (deeper green)	-	-	0.63	2.94	82%	8.0/1.8	20 cycles	140	[81]
**7**	5b	288	430 (10%)	430 (10%)	colorless	0.85 (greenish blue)	-	-	-	0.74	3.13	84%	-	100 cycles	186	[82]
**8**	5a	251	440 (10%)	439 (10%)	colorless	0.54 (orange-red)	0.75 (brilliant blue)	-	-	0.41	2.97	-	4.8/2.7 (1st) 4.6/2.0 (2nd)	1000 cycles	257 (1st) 278 (2nd)	[83]
**9**	6a	244	440	407	colorless	0.82 (green)	-	-	-	0.72	3.20	86%	2.5/0.6	100 cycles	268	[84]
**10**	SBF–HPA	-	>420	-	colorless	0.53 (green)	0.90 (blue)	-	-	0.37	-	65%	2.3/1.6 (1st) 1.9/3.2 (2nd)	200 cycles	246 (1st) 127 (2nd)	[85]
**11**	11–TPE–PA	247	-	-	colorless	0.77 (gray)	-	-	-	0.62	-	86%	2.2/0.8	300 cycles	-	[86]
	Z–12–TPE– PA	245	-	-	colorless	0.66 (black)	-	-	-	0.57	-	92%	0.6 s/0.3	300 cycles	-	
**12**	S–HPA	284	448	-	colorless	0.61 (orange red)	0.81 (blue)	-	-	-	-	64%	2.6/1.7	500 cycles	-	[87]
	P–HPA	317	-	-	yellow	0.77 (green)	(blue green)	-	-	-	-	48%	2.8/2.4	500 cycles	-	
**13**	a–HPA	251	-	-	colorless	0.62 (green)	1.03 (blue)	-	-	-	-	-	1.4/1.1 (1st) 1.8/1.2 (2nd)	500 cycles	296 (1st) 248 (2nd)	[88]
	b–HPA	241	-	-	colorless	0.65 (green)	1.03 (blue)	-	-	-	-	-	1.8/1.4 (1st) 2.2/1.1 (2nd)	500 cycles	197 (1st) 187 (2nd)	
**14**	PA–4	266	403	-	colorless	0.70 (green)	1.07 (blue)	-	-	-	-	69.2%	2.9/1.8	400 cycles	280	[89]
**15**	T3TPA–OA	-	321	-	yellow	dark green	-	-	-	−3.0	3.38	65%	4.3/3.7	12,000 s	206	[90]
**16**	SFXMOPb–OA	-	-	-	colorless	0.63 (red)	0.80 (blue)	-	-			62%	1.0/2.2	2500 cycles	463	[91]
**17**	PA– VIIID	315	448	-	light yellow	0.63 (green)	1.58 (dark blue)	-	-	0.3	2.97	58.7%	3.4/3.9 (1st) 3.9/3.9 (2nd)	200 cycles	203	[92]
**18**	P1	-	308	-	colorless	0.84 (blue-green)	-	-	-	0.66	3.25	22%	6.2/5.8	300 cycles	225	[93]
**19**	P2	-	-	-	colorless	1.00 (dark blue)	-	−1.17	-	0.78	2.20		6.69/2.29	500 cycles	219	[94]
**20**	TPABTA	-	-	295	reddish brown	1.6 (blue)	-	-	-	0.24	3.30	14%	3.2/4.4	-	141	[95]

^a^ Versus Ag/AgCl. E^1/2^: Oxidation half-wave potentials from cyclic voltammograms. ^b^ Oxidation initiation potential. ^c^ Calculated from the polymer films using the equation: E_g_ = 1240/λ_onset_ (energy gap between HOMO and LUMO). ^d^ T_c_: Timescale at oxidation state reaching 90% of the full switch in transmittance. T_b_: Timescale at reduction state reaching 90% of the full switch in transmittance. ^e^ Coloration efficiency is derived from the equation: η = ΔOD/Q while Q is Ejected charge, determined from the in situ experiments.

## Data Availability

Data are available in the source publications listed in the bibliography.
